# The relationship between study skills and depressive symptoms among medical residents

**DOI:** 10.1186/s12909-019-1870-x

**Published:** 2019-11-21

**Authors:** Eiad AlFaris, Muhannad AlMughthim, Farhana Irfan, Nassr Al Maflehi, Gominda Ponnamperuma, Huda E. AlFaris, Abdullah M. A. Ahmed, Cees van der Vleuten

**Affiliations:** 1 Department of Family and Community Medicine, King Saud University Chair for Medical Education Research and Development, College of Medicine, King Saud University, PO Box 2925, Riyadh 11461 Riyadh, Kingdom of Saudi Arabia; 2 Department of Family and Community Medicine, College of Medicine, King Saud University, Riyadh, Saudi Arabia; 3 College of Dentistry, King Saud University, Riyadh, Saudi Arabia; 4 Department of Medical Education, Faculty of Medicine, University of Colombo, Colombo, Sri Lanka; 5 Department of Internal Medicine, Prince Sultan Military Medical City, Riyadh, Saudi Arabia; 6 Department of Educational Development and Research Maastricht University, Maastricht, the Netherlands

**Keywords:** Medical residents, Study skills, Depressive symptoms

## Abstract

**Background:**

The cost of depression among residents is staggering as it extends into the quality and safety of patient care. Finding an explanation to resident depression by investigating the associated factors is therefore important. Study skills can be a possible factor, and a clear gap in the literature exists in this regard. We sought to investigate the relationship between depressive symptoms among residents and their study skills.

**Methods:**

This was a correlational study and a non-probability sample of 240 residents completed the Beck Depression Inventory II (BDI-II) and the Study Skills Inventory (SSI). Chi-square test was used to compare different categorical variables, while student t-test and ANOVA for continuous data. Pearson’s correlation coefficient was performed to find the relationship between depressive symptoms and study skills and the association that these measures have with the demographic variables.

**Results:**

Overall, 186 residents (76%) filled out the questionnaire. The SSI total score was found to have a significant negative association with the BDI-II depression score (Pearson correlation = − 0.447and *p* < 0.000). No significant association was found between the total SSI score and the residents’ sex, age, marital status, smoking status, training years or specialties.

**Conclusion:**

Poor study skills were found to be correlated with higher depressive symptoms. Future studies are required to develop a deeper understanding of this relationship and reconfigure the approach to study skills for the well-being of the future physicians.

## Background

Study skills (study strategies) are approaches to learning that help the processing of information acquisition [1]. They represent the conscious use of learning processes to get the best study practices [2]. Study skills cover a broad area of behaviours that learners can perform before, within and after the learning process to assist in processing and applying information [1].

To evaluate study skills of learners validated questionnaires such as LASSI (Learning and Study Strategies Inventory) [3], ASSIST (Approaches and Study Skills Inventory for Students) [4] and SSI (Study Skills Inventory) [5] are available.

Students often utilize ineffective study techniques such as cramming and highlighting textbooks [6]. Unfortunately, research studies show that this superficial approach to learning is due to excessive workload [7, 8]. Research also has shown that deep and strategic approach to learning is more effective, leading to better academic success and job fulfilment [9].

It is well-known that learners find it difficult to manage time and adapt their schedules to balance studying and other commitments [10]. Emotional management skills consist of the ability to maintain control when situation, people and events make excessive demands [11]. Unmanaged emotional reactions to stress could affect the learning process negatively [12].

However, to our knowledge, no comprehensive study on residents’ study skills has been reported, barring two studies on reading habits among surgical residents [13, 14].

Depression is a multi-faceted mental health disorder that affects a significant proportion of medical students and adversely affects all spheres of an individual’s inter-personal, social and occupational life [5]. Many scales are used in the screening for depression among adults. The most commonly used are Beck Depression Inventory II (BDI II), Center for Epidemiological Studies Depression Scale (CES-D), Hamilton rating scale for depression (HAM-D) [15]. The BDI has been used for 35 years to identify and assess depressive symptoms, and has been reported to be highly reliable regardless of the population.

Among medical students, the depression rate ranges from 8 to 70% [16, 17]. Postgraduate trainees (i.e. residents) have been found to have a high rate of depression [18]. A systematic review and meta-analysis of 31 cross-sectional studies and 23 longitudinal studies among resident physicians showed an overall pooled prevalence of depressive symptoms around 28.8%, ranging from 20.9 to 43.2% [1].

The effects of depression extend to the residents’ quality of life and academic attainment [19–26]. The adverse effects of depression are not limited to the individual only but may also affect the society at large in terms of quality of patient care, safety and professionalism [22, 27]. Unfortunately, the factors associated with residents’ depression remain unclear, and there is an apparent gap in the literature with regard to the relationship between the high prevalence of depressive symptoms among medical residents and its associated factors. Hence, there is a real need to better understand the associated factors, natural history and epidemiology of this debilitating illness. This should be the first step to delineate the possible preventive and therapeutic strategies to combat this ailment [22].

Research shows that efficient and effective study skills can help in both academic competence and success even in non-academic settings [10]. It has been also shown in a previous study that depressive symptoms are associated with low study skills among medical students [5]. Hence, one of the suggested possible factors associated with depression among residents may be deficient or weak study skills [25, 28]. Furthermore, identifying residents’ level of study skills is essential to support their learning and make their learning experience more enjoyable. However, there are no studies that explore the relationship between study skills and depressive symptoms among postgraduate medical trainees/residents in the literature. Depression is a prevalent and handicapping health problem that adversely affects all spheres of an individual’s and family life and in the case of healthcare professionals it extends to quality of patient care, safety and professionalism. Understanding the factors (like study skills) associated with depression is therefore of high importance.

This study aimed to explore the relationship between study skills and depressive symptoms among medical residents in different specialties and their relationship with demographic factors.

### Research objectives

1. To explore the relationship between study skills and depressive symptoms among medical residents.
2. To assess the relationship of demographic factors with the residents’ (a) study skills and (b) depressive symptoms.

## Methods

### Study design

Cross-sectional correlational study.

### Setting of the study

The study was conducted in King Saud University Medical City (KSUMC), Riyadh, Kingdom of Saudi Arabia, during the academic year 2017–2018. We distributed the inventories to 240 residents from the six major specialties (family medicine, internal medicine, obstetrics and gynecology [Ob-Gyn], general surgery, paediatrics and psychiatry) of all training years and both genders.

### Characteristics of the setting

The KSUMC is considered one of the largest postgraduate training centres in the Gulf region. It offers many postgraduate training programs such as family medicine, internal medicine, obstetrics and gynaecology (Ob-Gyn), general surgery, paediatrics, anaesthesia, emergency medicine, ophthalmology, orthopaedics, radiology, ENT and psychiatry. The duration of training varies from one to five years. Furthermore, currently, the KSUMC offers 28 fellowship programs.

### Data collection instruments

The following tools were administered for data collection:

1. **Demographic Questionnaire:** A short, six-item questionnaire was used to gather information about age, gender, smoking status, marital status, speciality and the training year.
2. **Study Skills Inventory (SSI):** This 23-item inventory was found to be both reliable and valid in a previous study among medical students [5].

The Study Skills Inventory (SSI) consists of five subscales namely reading skills, memory and concentration skills, time management, emotional management skills and other learning practices. Reading skill is the process of working with the text. In other words, readers negotiate the meaning with the author of the text that they read while applying their prior knowledge to it [7]. Both memory and concentration skills are part of multi-level cognitive abilities such as reasoning, problem-solving, and learning. Time management skill encompasses a set of techniques for managing and planning of different tasks and roles and appropriate use of time [29]. Emotional management skills are learned capabilities necessary to identify, evaluate, control emotions and develop adjustment capabilities for better educational achievements [30].

The maximum total score of SSI equals 69 (Table 6 in Appendix). Each statement has four options: always, usually, rarely and never.

This four-point scale is scored from 0 to 3; i.e.**3** stands for always (i.e. all the time - around 100% of the time), **2** stands for usually (i.e. sometimes - around 75% of the time), **1** stands for rarely (i.e. few times - around 25% of the time), **0** stands for never (i.e. almost at no times - around 0% of the time).

The scale has shown acceptable psychometric properties (i.e. validity and reliability) within the same country in the past [5, 31, 32].

3. Beck Depression Inventory (BDI-II):

This is a 21-item inventory, used to measure the severity of depression. Each item has four options and scored on a scale of 0 to 3. The maximum score for all items is 63, and the scores are divided into: minimal (0–13), mild (14–19), moderate (20–28), and severe depression (29–63). This scale is highly specific and sensitive for screening of depression with high reliability and validity [31].

The BDI-II has been used in many countries (including in the country of the present study) to measure depressive symptoms of both undergraduate and postgraduate medical students [32].

### Data collection

The three instruments: demographic questionnaire, Study Skills Inventory (SSI) and Beck Depression Inventory– II (BDI II) were distributed to the residents at the same time.

The covering page of the questionnaire explained the aims of the study and the participants were assured of anonymity and confidentiality. The participation was on a voluntary basis.

### Data analysis plan

The Statistical Package for the Social Sciences (SPSS, version 22) was used for the data analysis. Descriptive statistics such as frequency, percentages, mean, and standard deviation were calculated. Cronbach alpha coefficient was used to determine the reliability of the two measures; i.e. study skill scores and depression scores.

Comparisons were made using Chi square test to compare the following categorical variables (age, sex, smoking status, marital status, training year and speciality) and depressive symptoms’ severity (minimal, mild, moderate and severe). Association between total (and subscales) SSI mean scores and two-category variables (sex and marital status) were conducted using student’s t test. ANOVA was used for similar comparisons with variables having more than two categories (age, smoking status, training year, speciality and mean of study skills subscales).

Additionally, correlations were computed using Pearson’s correlation coefficient between the total depressive symptom (BDI -II) scores and the study skills inventory (SSI) scores.

After finding a relatively low reliability for the BDI-II, we applied a statistical correction test called “an attenuation correction” [33] to the correlation coefficient between SSI and BDI-II.

The observed correlation is divided by the square rooted product of the reliabilities of both tests.

### Ethics approval and consent to participate

Informed consent was obtained from all residents prior to data collection. All the selected respondents were given assurance of confidentiality that the information gathered will be used exclusively for research purposes. This study was approved by the Institutional Review Board of the College of Medicine; KSU (reference no: 17/0330/IRB).

## Results

Overall, 186 residents filled out the SSI, BDI-II and demographic questionnaire (response rate of 76%). Family medicine residents represented 31.7% of those who responded, followed by, internal medicine (28%), peadiatrics (18.3%), general surgery (9.1%), obstetrics-gynaecology (6.5%) and psychiatry residents (6.5%).

Cronbach alpha reliability coefficients of SSI and BDI-II were 0.75 and 0.58 respectively. The correlation coefficient between SSI and BDI-II (before the attenuation correction) was - 0.296. The correlation value Rxy = − 0.296, the reliability of SSI is 0.749 (Rxx) and reliability of BDI is 0.584. Hence the Rtrue = − 0.296/Square root of (0.749)(0.584) = − 0.447. The true correlation after applying the attenuation correction was found to be - 0.447.

### Participants’ demographic characteristics

Out of 186 respondents, 99 (53.2%) were female. The majority were single (59.7%) and never smoked (76.3%) (Table 1). The mean age of the participants was 26.9 years.

**Table 1:** Distribution of residents in terms of socio-demographic characteristics

Socio-demographic data	Number (%) of residents
Sex	
Male	87 (46.8)
Female	99 (53.2)
Age	
23–26 years	77 (41.4)
27–30 years	101 (54.3)
> 30 years	8 (4.3)
Marital status	
Single	111 (59.7)
Married	73 (39.2)
Divorced	2 (1.1)
Smoking status	
Never smoker	142 (76.3)
Current smoker	36 (19.4)
Ex-smoker	8 (4.3)
Training year	
1st year	48 (25.8)
2nd year	35 (18.8)
3rd year	66 (35.5)
4th year	29 (15.6)
5th year	6 (3.2)
Additional year	2 (1.1)
Specialty	
Family medicine	59 (31.7)
Internal medicine	52 (28)
Pediatrics	34 (18.3)
General surgery	17 (9.1)
OB-Gyne	12 (6.5)
Psychiatry	12 (6.5)

The mean ± standard deviation of the total SSI score was 42.56 ± 8.06 (62% out of a maximum 69), while the mean ± standard deviation of depressive symptoms score was 11.64 ± 10.09 (18.4% out of a maximum 63). The mean ± standard deviation for the SSI score for the subscales are shown in Table 2.

**Table 2:** Mean (±SD) of study skills subscales

Study skills subscales	Mean (±SD)	Maximum Score	% out of the maximum
Reading skills	8.58 (± 1.84)	12	71.5
Concentration and memory skills	3.43 (±.96)	6	57.1
Time management skills	13.24 (± 4.17)	24	55.1
Emotional management skills	8.07 (± 2.14)	12	67.2
Other learning skills	9.24 (± 2.63)	15	61.6

There was a statistically significant moderate negative correlation between BDI-II score and the SSI total score (r = − 0.447, *p* < 0.001). However, no significant association was found between the total SSI score and sex, age, marital status, smoking status, training years or specialties (Table 3).

**Table 3:** Association between total SSI score and socio-demographic characteristics

Socio-demographic characteristics		N	Total SSI scores mean (±SD)	F ratio / t statistic^*^	*p*-value^*^
Sex	Male	87	42.25 (±7.86)	0.164	0.686
	Female	99	42.84 (±8.27)		
Age	23–26	77	43.66 (±7.86)	1.222	0.297
	27–30	101	41.80 (±8.32)		
	> 30	8	41.63 (±5.98)		
	Total	186	42.56 (±8.06)		
Marital status	Single	111	43.14 (±8.4)	0.721	0.378
	Married	73	42.08 (±7.25)		
Smoking status	Never smoker	142	42.82 (±8.06)	0.672	0.512
	Current smoker	36	41.25 (±8.61)		
	Ex-smoker	8	44 (±5.1)		
	Total	186	42.56 (±8.1)		
Training year	1st year	48	42.96 (±7.1)	1.45	0.209
	2nd year	35	45.37 (±8.92)		
	3rd year	66	40.95 (±7.99)		
	4th year	29	42.28 (±8.04)		
	5th year	6	41.67 (±10.23)		
	additional year	2	44 (±2.83)		
	Total	186	42.56 (±8.06)		
Specialty	Family Medicine	59	43.58 (±8.43)	1.017	0.409
	Internal Medicine	52	40.85 (±7.54)		
	General Surgery	17	42.88 (±8.33)		
	Pediatrics	34	44.09 (±7.47)		
	OB-Gyne	12	41 (±10.05)		
	Psychiatry	12	41.83 (±7.51)		
	Total	186	42.56 (±8.06)		

*ANOVA was used for all the associations, except for the sex and marital status, the T test was used

For the study skills domains, a statistically significant association was found between the student sex (in favour of male students) and the concentration and memory skills (*p* = 0. 004) (Table 4). Furthermore, time management had statistically significant associations with age (*p* = 0.007) and training years (*p* = 0.035) but no other consistent trends were found with both age or training years). (Table 4). There were no significant associations between the different study skills domains and the residents’ specialty except emotional management skills (*p* = 0.037). This latter domain was higher among family medicine and paediatric residents compared to obstetrics-gyneacology (Ob-Gyn), and internal medicine residents. (Table 4). The rate of depression was higher among the female residents (39%) than their male counterparts (19%), and the difference was statistically significant (*p* = 0.007) (Table 5).

**Table 4:** Association between study skills subscales and socio-demographic characteristics

Socio-demographic characteristics		N	Mean of study skills subscales (±SD)				
			Reading skills	Concentration and memory skills	Time management skills	Emotional management skills	Other learning skills
Sex	Male	87	8.37 (**±**1.98)	3.64 (**±**.94)	12.81 (**±**3.98)	8.16 (**±**2.09)	9.25 (**±**2.19)
	Female	99	8.74 (**±**1.70)	3.24 (**±**.95)	13.61 (**±**4.32)	8 (**±**2.20)	9.23 (**±**2.98)
	t-statistic (*p*-value)		0.175	0.004	0.193	0.611	0.958
Age	23–26	77	8.42 (**±**1.74)	3.44 (**±**.97)	14.36 (**±**3.68)	8.35 (**±**2.01)	9.07 (**±**2.65)
	27–30	101	8.74 (**±**1.96)	3.42 (**±**.97)	12.40 (**±**4.17)	7.95 (**±**2.29)	9.27 (**±**2.68)
	> 30	8	7.87 (**±**.99)	3.37 (**±**.74)	13 (**±**6.26)	7 (**±**.75)	10.37 (**±**1.59)
	Total	186	8.57 (**±**1.84)	3.43 (**±**.96)	13.24 (**±**4.17)	8.07 (**±**2.14)	9.24 (**±**2.63)
	F-ratio (*p*-value)		0.292	0.981	0.007	0.164	0.41
Marital status	Single	111	8.63 (**±**1.90)	3.46 (**±**.93)	13.56 (**±**4.18)	8.20 (**±**2.13)	9.26 (**±**2.76)
	Married	73	8.49 (**±**1.79)	3.38 (**±**1.02)	12.94 (**±**4.04)	7.95 (**±**2.14)	9.30 (**±**2.43)
	t-statistic *p*-value		0.45	0.483	0.901	0.365	0.271
Smoking status	Never smoker	142	8.59 (**±**1.77)	3.44 (**±**.97)	13.30 (**±**4.01)	8.19 (**±**1.97)	9.29 (**±**2.78)
	Current smoker	36	8.39 (**±**2.12)	3.36 (**±**1.02)	12.78 (**±**4.61)	7.64 (**±**2.76)	9.08 (**±**2.29)
	Ex-smoker	8	9.13 (**±**1.80)	3.63 (**±**.51)	14.25 (**±**5.14)	8 (**±**2.14)	9 (**±**1.19)
	Total	186	8.57 (**±**1.84)	3.43 (**±**.96)	13.24 (**±**4.17)	8.08 (**±**2.15)	9.24 (**±**2.63)
	ANOVA *p*-value		0.583	0.773	0.627	0.388	0.88
Training year	1st	48	8.31 (**±**1.65)	3.27 (**±**.68)	14.25 (**±**3.67)	8.33 (**±**2.18)	8.79 (**±**2.58)
	2nd	35	8.77 (**±**1.91)	3.66 (**±**1.1)	14.4 (**±**4.49)	8.37 (**±**1.89)	10.17 (**±**2.57)
	3rd	66	8.36 (**±**1.93)	3.45 (**±**1.01)	12.48 (**±**4.26)	7.77 (**±**2.08)	8.88 (**±**2.45)
	4th	29	9.03 (**±**1.91)	3.45 (**±**1.06)	11.93 (**±**3.49)	8.38 (**±**2.09)	9.48 (**±**3.12)
	5th	6	9.67 (**±**1.37)	2.83 (**±**.75)	12.17 (**±**5.53)	6.83 (**±**3.71)	10.16 (**±**2.14)
	additional year	2	8.5 (**±**.71)	4 (**±**1.41)	16 (**±**4.24)	6(**±**1.41)	9.5 (**±**2.12)
	Total	186	8.57 (**±**1.84)	3.43 (**±**.96)	13.24 (**±**4.17)	8.07 (**±**2.15)	9.24 (**±**2.63)
	ANOVA *p*-value		0.294	0.278	0.035	0.211	0.154
Specialty	Family Medicine	59	8.49 (**±**1.96)	3.46 (**±**.88)	13.24 (**±**4.26)	8.73 (**±**2.17)	9.66 (**±**2.42)
	Internal Medicine	52	8.38 (**±**1.75)	3.5 (**±**.80)	12.88 (**±**4.09)	7.62 (**±**2.09)	8.46 (**±**2.76)
	General Surgery	17	8.06 (**±**1.89)	3.65 (**±**1.06)	13.88 (**±**3.49)	7.94 (**±**2.01)	9.35 (**±**2.42)
	Pediatrics	34	8.85 (**±**1.73)	3.35 (**±**1.07)	14.12 (**±**4.21)	8.21 (**±**1.84)	9.56 (**±**2.55)
	OB-Gyne	12	9.58 (**±**2.19)	3.08 (**±**1.08)	12.17 (**±**4.89)	6.92 (**±**3.18)	9.25 (**±**2.18)
	Psychiatry	12	8.75 (**±**1.36)	3.25 (**±**1.42)	12.5 (**±**4.36)	7.83 (**±**1.11)	9.5 (**±**3.68)
	Total	186	8.57 (**±**1.84)	3.43 (**±**.96)	13.24 (**±**4.17)	8.08 (**±**2.15)	9.24 (**±**2.63)
	ANOVA *p*-value		0.264	0.647	0.635	0.037	0.245

**Table 5:** Association between depressive symptoms’ severity and sex

Sex	Depressive symptoms severity				Total N (%)	*p*-value^*^ (X^2^ value)
	Minimal N (%)	Mild N (%)	Moderate N (%)	Severe N (%)		
Male	70 (80.5%)	7 (8%)	8 (9.2%)	2 (2.3%)	87 (100%)	.007 (12.025)
Female	60 (60.6%)	8 (8.1%)	18 (18.2%)	13 (13.1%)	99 (100%)	
Total N (%)	130 (69.9%)	15 (8.1%)	26 (14%)	15 (8.1%)	186 (100%)	

*The Chi- square statistical test was used for the association between the categorical variables

There was also a statistically significant association between the training year and the severity of depression, the senior residents had more severe depression compared to their junior counterparts (*p* = 0.026).

## Discussion

This study found that residents’ study skills were negatively associated with depressive symptoms. To the best of our knowledge, this is the first time that the relationship between study skills and depressive symptoms has been reported among residents or postgraduate medical trainees.

Female residents were more likely to be depressed than their male counterparts. This is in line with other studies [32]. The current study revealed that there was a non-significant difference in study skills between the two sexes. This is also in agreement with other studies conducted among medical students [7, 10, 34].

The present study found that senior residents are more likely to report depressive symptoms than their junior counterparts. A meta-analysis of 54 studies on physicians in training in different specialties showed that the overall pooled prevalence of depression or depressive symptoms was 28.8% and ranged from 20.9 to 43.2%, depending on the instrument used, and increased positively with residency year (upper-level residents had a higher proportion of depressive symptoms) [1]. Spending too many hours at work and multitasking put a lot of cognitive load. Literature reports these, which indirectly relate to time management, as contributors to burnout [35].

In this study too, time management showed a significant association with both years of training and age.

The emotional management scores were significantly different among the residents of different specialties in our study. The unpredictability of work, patient care, interruptions, and variable work schedules are usually uncontrollable from speciality to speciality [35]. Residents from different specialities may have developed varying strategies to cope with these uncontrollable factors depending on their speciality. This may explain the different emotional management skills reported by the residents of different specialities.

Well-being on the other hand is multifaceted, as it is not only performing well on standardized tests but performing and excelling in life too. Previous research showed that there was a positive relationship between wellbeing and academic achievement [36, 37]. Poor study skills were shown clearly to reduce academic achievements that may lead to emotional exhaustion, and difficulty in coping the stresses of residency training and suboptimal quality of life [13, 14, 35]. This will ultimately affect personal as well as patient care in the long run [1]. Therefore, more efforts should be directed to improve study skills in addition to better understanding the factors related to it.

### Limitations

The low reliability of the BDI II 0.584 could be explained by the relatively small sample size in this study. It had shown a much higher reliability in many countries [38]. As the poor reliability decrease correlation coefficient and with the statistical attenuation correction, the actual correlation coefficient was found to be stronger. As this study is correlational in nature, causality cannot be claimed. Also, since the study did not take into account the training culture and educational environments of the residents, how these factors impact on depressive symptoms and study skills could not be elucidated. Since multiple factors affect both study skills and depression, this study should be viewed as an investigation of two multi-factorial constructs, without controlling for the other known and unknown factors. The moderate correlation coefficient found in this study could hence be explained by the inability of a study of this nature to account for all such confounding factors that may affect both study skills and depression.

We also acknowledge that SSI has not been used to measure study skills among postgraduate students previously. However, the acceptable reliability of SSI and the significantly negative moderate correlation of SSI with depressive symptoms observed in this study validate the use of SSI among postgraduates.

Another limitation is this study was conducted in a single institution, which may reduce the generalizability of the results. Therefore, we need a multi-centre prospective study to provide a better understanding of this topic.

## Conclusions and recommendations

The results of this study suggest that depressive symptoms and study skills show a moderate, negative relationship. Additional studies are needed to establish whether poor study habits and lower academic performance contribute to depression or whether depression leads to poor study habits such as surface learning.

Implementing a course for a high level of study skills among residents may help them to improve their mental health. This is especially so, if later studies find that poor study skills are one of the contributing factors for depressive symptoms among residents.

## Data Availability

The data that support the findings of the current study are available from the corresponding author on reasonable request.

## Appendix

**Table 6:** Study Skills Inventory (SSI)

	Reading text	Always	Usually	Rarely	Never
1.	I try to organize facts in a systematic way.				
2.	I look for the main ideas as I read.				
3.	I take notes as I read my textbooks.				
4.	When reading, I highlight important passages.				
	Concentration and memory	Always	Usually	Rarely	Never
5.	I study even when less important things distract me				
6.	I give full attention to the tasks.				
7.	I keep up with assignments, readings and tests preparations, avoiding any delays				
8.	I keep study time a priority, saying “no” to social demands and extracurricular events				
9.	I avoid activities, which tend to interfere with my planned schedule.				
	Time management	Always	Usually	Rarely	Never
10.	At the beginning of the term, I make up my activity and study schedules				
11.	I break assignments into manageable parts.				
12.	I use a “to do” list to keep track of the tasks.				
13.	I plan my day by deciding what is important to do				
14.	I outline specific goals for my study time.				
	Emotional management	Always	Usually	Rarely	Never
15.	I plan regular times for fun.				
16.	I take relaxation or rest time when under stress.				
17.	I regularly try to motivate myself to keep up with the planned schedule				
18.	I try to get rid of negative thoughts and worrying while studying				
	Other learning practices	Always	Usually	Rarely	Never
19.	When I do not understand something, I get help from classmates				
20.	I participate in class discussions.				
21.	I volunteer answers to questions posed by instructors in the class.				
22.	I see learning as something I will be doing all throughout my life.				
23.	I ask the instructor questions when clarification is needed.				

## Abbreviations

BDI II: Beck Depression Inventory II
Ob-Gyn: Obstetrics and Gynecology
SSI: Study skills inventory
